# Transforming Orthopedic Joint Surgeries: The Role of Artificial Intelligence (AI) and Robotics

**DOI:** 10.7759/cureus.43289

**Published:** 2023-08-10

**Authors:** Sazid Hasan, Ashar Ahmed, Muhammad A Waheed, Ehab S Saleh, Abdullah Omari

**Affiliations:** 1 Orthopedic Surgery, Oakland University William Beaumont School of Medicine, Rochester, USA; 2 Biology, Wayne State University, Detroit, USA; 3 Orthopedics, Wayne State University, Detroit, USA; 4 Orthopedic Surgery, Beaumont Hospital, Royal Oak, USA

**Keywords:** joints, surgical advances, surgical robotics, ai and machine learning, ai & robotics in healthcare

## Abstract

The landscape of orthopedic joint surgeries, specifically total hip arthroplasty (THA) and total knee arthroplasty (TKA), is rapidly changing, and artificial intelligence (AI) along with robotics is at the helm of this transformation. These technologies, working synergistically, have introduced unprecedented levels of precision and personalization to surgical procedures, thereby significantly enhancing patient outcomes. In this editorial, we explore the changing perspectives of orthopedic surgeons toward AI and robotics and dissect the incorporation of these technologies in surgeries, their associated advantages, their inherent limitations, and potential future prospects. We draw from a host of recent studies to provide a comprehensive understanding of how these transformative technologies can augment surgical performance and patient care.

## Editorial

The changing perceptions in orthopedic surgery

The perception toward artificial intelligence (AI) and robotics among orthopedic surgeons is on a positive trajectory. Far from being viewed as mere adjuncts, these technologies are increasingly recognized as catalysts that can elevate surgical precision and patient outcomes. This is a significant step towards the realization of the tenets of personalized medicine [1-3]. An example is robotic systems like Stryker's Mako. These systems provide orthopedic surgeons with preoperative CT-based 3D modeling for surgical planning, and real-time intra-operative guidance, enabling real-time adjustments and precise alignment of implants, thereby paving the way for optimized patient outcomes.

Consider the study by Houserman et al., where an AI model was utilized to predict the suitability of patients for total knee arthroplasty (TKA), unicompartmental knee arthroplasty, or no surgical intervention [1]. This model holds the potential in enabling surgeons to tailor surgical decisions according to individual patient needs. It can aid in reducing unnecessary surgeries and optimizing healthcare resources, thereby significantly impacting patient care.

Similarly, Wu et al. developed an AI-based 3D preoperative planning system (AIHIP) for total hip arthroplasty (THA) [2]. This system was found to provide a more accurate surgical planning tool compared to traditional acetate templating. By enabling precise visualization of patient-specific anatomical structures, the AIHIP system has shown promise in enabling better surgical execution, potentially leading to fewer postoperative complications and improved patient outcomes.

The emergence of state-of-the-art technology and procedures

AI and robotics together have ushered in a new era in orthopedic surgery. Machine learning (ML) algorithms are increasingly being used for diagnosis, prognostication, and surgical planning, significantly impacting patient care [3]. These algorithms have demonstrated remarkable predictive prowess, allowing for the forecasting of surgical outcomes, and thereby guiding surgeons in tailoring surgical interventions for enhanced patient satisfaction.

The use of AI models to automate preoperative planning for orthopedic surgeries is a notable advancement [4]. As Lambrechts et al. demonstrated, by using ML to automate patient- and surgeon-specific preoperative planning in patient-specific joint replacement, surgical planning accuracy can be improved significantly. It also considerably reduces the time surgeons need for preparation, enhancing overall surgical efficiency, and possibly leading to better patient outcomes.

Interpreting current data and outcomes

Recent studies provided compelling evidence supporting the role of AI and robotics in orthopedic joint surgeries. These technologies have been associated with improved patient outcomes, including enhanced satisfaction and reduced complications. These benefits are largely attributable to the high degree of precision that these technologies bring to surgical procedures [1,3].

The AIHIP system, for instance, has shown significantly improved surgical outcomes, particularly in terms of providing greater detail and accuracy in visualizing patient anatomical structures compared to traditional 2D planning systems [2]. This strongly suggests the growing potential of 3D AI-based systems in orthopedic surgery.

Advances in AI and robotics

The role of AI and robotics in orthopedic joint surgeries continues to expand, with ongoing advances pushing the boundaries of what is possible in surgical practice. AI models are being used, for instance, to predict patient suitability for different types of knee surgeries, showcasing their valuable role in guiding surgical decisions [1,5-9].

Furthermore, recent literature, as demonstrated by Helm et al., highlighted that the incorporation of ML into orthopedics has the potential to elevate patient care through alternative patient-specific payment models, rapidly analyze imaging modalities, and remotely monitor patients [8]. These emerging applications of AI warrant ownership, leverage, and application by orthopedic surgeons to better serve their patients and deliver optimal, value-based care.

In addition to prediction, AI models are being used to streamline preoperative workflows, a significant development that holds considerable promise [4,9]. With these tools, preoperative planning can be made significantly more efficient, requiring fewer manual corrections by surgeons and thus freeing up their time for other important tasks. This advancement in AI applications is an excellent example of how AI can improve the workflow in a surgical setting, thereby enhancing patient outcomes and overall healthcare efficiency.

Limitations of AI and robotics

Despite the significant strides made, challenges persist in the full integration of AI and robotics into orthopedic surgery. The limitations include issues related to data quality and accessibility, the cost of implementation, the learning curve associated with these technologies, and the need to strike a balance between human expertise and technological assistance [5]. Addressing these challenges systematically will be essential for realizing the full benefits of AI and robotics in orthopedic surgery.

Future directions in AI and robotics

Future research is focusing on refining AI algorithms to predict long-term implant survivorship, patient-reported outcomes, and overall functional recovery after surgery. The role of AI in predicting patient satisfaction post-surgery is especially intriguing, given the current emphasis on patient-centered care in modern medicine [3].

Moreover, the convergence of AI with other technologies such as virtual and augmented reality could revolutionize surgical procedures. For instance, the integration of AI algorithms for autonomous decision-making during surgery could lead to more precise and efficient procedures [6].

In conclusion, AI and robotics are playing a pivotal role in the evolution of orthopedic joint surgeries. While the role of surgeons remains indispensable, AI and robotics serve to augment their capabilities, enhancing patient outcomes and satisfaction. However, there are challenges that need to be addressed to fully harness these advancements. Looking forward, the future of orthopedic surgery is ripe with opportunities as we continue to refine and explore these technologies.
